# Major pathways involved in macrophage polarization in cancer

**DOI:** 10.3389/fimmu.2022.1026954

**Published:** 2022-10-17

**Authors:** Clément Kerneur, Carla E. Cano, Daniel Olive

**Affiliations:** ^1^ ImCheck Therapeutics, Marseille, France; ^2^ Team Immunity and Cancer, Centre de Recherche en Cancérologie de Marseille (CRCM), Inserm U1068, CNRS UMR7258, Institut Paoli Calmettes, Marseille, France

**Keywords:** signaling/signaling pathways, cancer biology, TAMs (tumor associated myeloid cells), TME (tumor microenvironment), macrophages polarization

## Abstract

Macrophages play an important role in tissue homeostasis, tissue remodeling, immune response, and progression of cancer. Consequently, macrophages exhibit significant plasticity and change their transcriptional profile and function in response to environmental, tissue, and inflammatory stimuli resulting in pro- and anti-tumor effects. Furthermore, the categorization of tissue macrophages in inflammatory situations remains difficult; however, there is an agreement that macrophages are predominantly polarized into two different subtypes with pro- and anti-inflammatory properties, the so-called M1-like and M2-like macrophages, respectively. These two macrophage classes can be considered as the extreme borders of a continuum of many intermediate subsets. On one end, M1 are pro-inflammatory macrophages that initiate an immunological response, damage tissue integrity, and dampen tumor progression by fostering robust T and natural killer (NK) cell anti-tumoral responses. On the other end, M2 are anti-inflammatory macrophages involved in tissue remodeling and tumor growth, that promote cancer cell proliferation, invasion, tumor metastasis, angiogenesis and that participate to immune suppression. These decisive roles in tumor progression occur through the secretion of cytokines, chemokines, growth factors, and matrix metalloproteases, as well as by the expression of immune checkpoint receptors in the case of M2 macrophages. Moreover, macrophage plasticity is supported by stimuli from the Tumor Microenvironment (TME) that are relayed to the nucleus through membrane receptors and signaling pathways that result in gene expression reprogramming in macrophages, thus giving rise to different macrophage polarization outcomes. In this review, we will focus on the main signaling pathways involved in macrophage polarization that are activated upon ligand-receptor recognition and in the presence of other immunomodulatory molecules in cancer.

## Introduction

### Tumor Associated Macrophages can be polarized into pro-inflammatory (M1-like) and anti-inflammatory (M2-like) phenotypes

#### Tumor-Associated Macrophages

TME is the result of complex interactions between different cell types, including tumor cells, immune cells, endothelial cells, and fibroblasts. TAMs, which represent 50–80% of non-tumor stromal cells, are critical components of the TME. After recruitment of circulating monocytes *via* the chemokine ligand 2 (CCL2)- chemokine-receptor 2 (CCR2) axis and many others chemokines and cytokines such as macrophage colony-stimulating factor (M-CSF), chemokine (C-X3-C motif) ligand 1 (CX3CL1), CCL3, CCL5, and vascular endothelial growth factor A (VEGF-A) (1), TME will favor their polarization either towards anti-inflammatory or pro-inflammatory macrophages, that will be referred to as M1-like or M2-like TAMs, respectively. The two main macrophage polarization states described above and detailed below constitute most of the TAMs compartment, with a large predominance of the M2-like phenotype. However, this TAMs dichotomy is a simplistic way to represent their complex functions in the microenvironment. Recent data obtained using techniques such as single cell RNA sequencing may contribute to better identify macrophage subpopulations and have shown previously unknown complexity in macrophage polarization, going much beyond the traditional dogma of the binary “M1-M2” system (2–4). Nevertheless, regarding the fact that the categorization of tissue macrophages in inflammatory situations remains difficult and variable among different indications and patients, there is an agreement that TAMs are predominantly polarized into the two main subtypes, M1-like and M2-like TAMs. Indeed, TAMs will change from one type to another depending on the environment in which they reside further described in next sections (1.b. and 1.c.).

In addition, it has been well established that TAMs prevalence in many solid tumors, such as breast, ovarian, cervical, and prostate cancers, is associated with poor prognosis, tumor progression, and metastasis (5–7). Indeed, TAMs promote tumor development by several ways:

- Anti-inflammatory TAMs control immune responses by secreting immunosuppressive cytokines and enzymes resulting in promotion of tumor growth and survival (8). In addition, TAMs also impair tumor-infiltrating lymphocytes’ (TILs’) capacity to migrate and interact with malignant cells (9).
- TAMs also enhance cancer stem cell-like properties of tumor cells (10) that promote invasive capability and metastasis of later stages cancers including formation of the pre-metastatic niche in secondary site, extravasation, and early colonization (11, 12) and favors angiogenesis by secreting proangiogenic factors including, placental growth factor (PIGF), TGF-β, CCL2, and CXCL12 (13–15).
- Furthermore, TAMs contribute to cancer resistance and relapse after treatment in radiotherapy and with several chemotherapeutic agents *via* releasing survival factors leading to malignant cells activation of anti-apoptotic signaling pathways (16–18). Additionally, direct contact of myeloma cells with macrophages can also induce chemoresistance (19). Indeed, relapse from anticancer treatment can be due to TAMs polymorphism during tumor development (20), for example in breast cancer treated with Taxol (21) or hepatocellular carcinoma treated with Sorafenib (22) both *in vitro* and *in vivo* (mice model).

Targeting TAMs could thus be an effective and promising therapeutic strategy for enhancing anti-cancer immunity. Current development of potentially but not yet effective medicines that target TAMs are based on TAM depletion, enhancement of TAM phagocytosis, inhibition of monocyte recruitment, and reprogramming of anti-inflammatory M2-like TAMs into pro-inflammatory M1-like TAMs. Therefore, it is essential to understand better the molecular and biological mechanisms leading to macrophage recruitment and polarization in cancer, and which functions are impacted.

#### Pro-inflammatory Macrophages (M1-like)

IFN-γ was the first macrophage-activating factor discovered in 1970 (23). IFN-γ is a soluble cytokine produced by activated CD4+ T helper (Th) 1 cells, CD8+ T cytotoxic cells, and NK cells that drives resting macrophages into potent cells with enhanced antigen–presenting capacity, increased synthesis of proinflammatory cytokines and toxic mediators, and enhanced complement–mediated phagocytosis among other functions. This early description of macrophage activation came to be known as classical activation. Since classically activated macrophages result from Th1 (cytotoxic) T cell responses, these macrophages were therefore renamed M1 macrophages. As well as IFN-γ, M1 macrophages can be differentiated by other Th1 cytokines, like TNF-α, and bacterial components such as lipopolysaccharide (LPS). Granulocyte-macrophage colony stimulating factor (GM-CSF) also activates M1 responses and antitumoral activity (17). Conversely, M1 macrophages stimulate the response of Th1 and cytotoxic cells, which in turn release IFN-γ, thereby reinforcing M1 polarization in a feedback loop (23–26). M1 macrophages release significant amounts of pro-inflammatory cytokines including IL-1β, IL-6, IL-12, IL-23, IFN-β and TNF-α that concomitantly drive Th1 responses (27, 28). M1 macrophages are also a main source of IL-12, while producing little or no IL-10, further amplifying M1 polarization.

The chemokines produced by M1 macrophages are mostly of the CC type (CCL2, CCL3, CCL4, CCL5, CCL8, CCL19, CCL20), moreover IFN-γ also increases the production of CXCL10 and CXCL9 leading to IFN-β expression. Overall, these chemokines contribute to the establishment of a type 1 immune response (29), resulting in protection against intracellular pathogens and tumor progression.

It is crucial to highlight that pro-inflammatory M1 macrophages can act as tumor suppressors in many ways:

- Destroying malignant cells. Pro-inflammatory Macrophages produce reactive oxygen species (ROS) and enhance their production by tumor cells through secretion of stimuli like TNF-α. ROS production by macrophages as a mean of killing tumor cells is well documented. Macrophages rapid generation of superoxide is driven predominantly by NAPDH oxidase resulting in hydrogen peroxide synthesis (30). In addition, during inflammation, nitric oxide (NO) synthetized by the inducible nitric oxide synthase (iNOS) from L-arginine reacts with superoxide to produce peroxinitrite radicals that are similar in their activity to hydroxyl radicals, and contribute to direct tumor cell apoptosis (31, 32). M1 also play an important role in tumor suppression through the generation of cytotoxic molecules such as TNF-α-related apoptosis inducing ligand (TRAIL) or FAS ligand (FASL), or *via* the Antibody-Dependent Cellular Phagocytosis (ADCP) or Antibody-Dependent Cell-mediated Cytotoxicity (ADCC) (24, 25).
- Stimulating anti-tumor immunity. The crosstalk between NK cells and macrophages constitutes a line of defense against tumors. Indeed, M1 macrophages express CD48, which binds to the 2B4 receptor at the plasma membrane of NK cells inducing IFN-γ production, which in turn supports M1 phenotype. In addition, release of IL-1β, IFN-β, and/or IL-23 by M1 will promote the expression of natural killer cell p44-related protein (NKp44) and natural killer group 2 member D (NKG2D). Consequently, NK cells will carry cytotoxic responses against target cells that express their ligands, such as stress ligands (33, 34). These cells are also able to activate CD8+ cytotoxic T cells (35), which are the most potent anticancer immune effectors and constitute the foundation of current effective cancer immunotherapies (36) through the secretion of the chemokines previously listed (CCL2, CCL3, CCL4, CCL5 and CXCL10) (37). M1 macrophages also express major histocompatibility complex (MHC)-II molecules and CD80/CD86 costimulatory molecules that are essential for T-cell activation, cytokine production, proliferation, and differentiation (38–41). M1-secreted CCL2, CCL5 or IFN-β may increase neutrophil infiltration to the tumor location and contribute to pro-inflammatory neutrophil-targeted tumor regression (42, 43).

Several reports indicate that the highest the M1/M2 ratio, the longest the survival in cancer patients. Indeed, lower proportion of M2-like TAMs in the TME enhances the prognosis of patients in multiple different cancer indications such as ovarian cancer (44, 45), multiple myeloma (46), pediatric classical Hodgkin lymphoma (47), colorectal cancer (CRC) (48) and gastric cancer (49). M1-like TAMs are also correlated with better efficacy of currently used chemotherapy in ovarian and lung cancer (50, 51). M1-like abundancy also leads to a tumor immunological status which, when combined with chemotherapy, aids in tumor growth control (50).

#### Anti-inflammatory Macrophages (M2-like)

In contrast to M1, M2 macrophages have anti-inflammatory and tumor promoting properties. M2-like TAMs predominate in advanced tumors, functioning as critical regulators in response to TME. Several studies have shown that M2-like macrophages represent most of TAMs in many cancer indications, which is associated with poor prognosis in several cancer patients, consistent with their abilities to favor tumor growth, to promote cancer invasion and metastasis, to participate in neovascularization, and to contribute to the formation of the immunosuppressive TME (25, 52–63).

M2 macrophages were first found in the setting of helminth infection, distinguishing from monocytic progenitors under the influence of IL-4 and IL-13 in a severely Th2-polarized response. Although Th2 cytokines are required for M2 recruitment, M2 macrophages also secrete cytokines that mediate Th2 responses and regulate inflammatory responses (64–67).

The primary agents that cause M2 type activation include cytokines (IL-4, IL-10, IL-34 and IL-13), vitamin D3, TGF-β, prostaglandin E2 (PGE2), VEGF, EGF, glucocorticoids (68) and M-CSF (69). M2-like TAMs are further sub-categorized into IL-4/13–activated M2a, immune complex–activated M2b, IL-10–deactivated M2c and IL-6/M-CSF loop induced M2d (70) macrophages based on the stimulation of different cytokines. Those subtypes will not be discussed here but are well described in a review by Mantovani et al. (29) and Duluc et al. (70).

M2 macrophages act as tumor promoters in several ways:

- Promoting cancer cell proliferation and invasion. EGF secreted by M2-like TAMs directly stimulates cancer cell proliferation and invasion. M2 macrophages can also contribute directly to cell proliferation, invasiveness, and migration in breast cancer by suppressing IFN regulatory factor (IRF)7 expression through MicroRNAs mIR-1587 (71). Moreover, several studies have shown that microRNAs play critical roles in macrophage activation, polarization, tissue infiltration, and inflammation resolution (miR-155, miR-181, and miR-451 are implicated in M1 macrophage polarization whereas miR-146a, miR-125a, and miR-145-5p are involved in M2 macrophage polarization) (72).
- In contrast to M1-like macrophages, NO and iNOS production appears diminished after TAMs switch to an M2-like phenotype leading to abolishment of M1 direct killing of tumor cells (73).
- Promoting angiogenesis. VEGF secretion by M2-like TAMs promotes angiogenesis in tumor sites. In turn, VEGF receptors on the surface of TAMs activate an autocrine loop, reinforcing their pro-angiogenic and immunosuppressive properties (25) but also their M2 phenotype. TAMs also secrete pro-angiogenic chemokines (e.g., IL-8) and proteolytic enzymes (e.g., MMPs and cathepsins induced by IL-4) (74), which breakdown the extracellular matrix (ECM), causing the release of angiogenic components such as TGF-β, VEGF, and fibroblast growth factor (FGF) that had previously been stored in the ECM in an inactive state (75–77). MMP-2 and MMP-9, were also shown to increase VEGF expression by cancer cells (76), and their expression has been linked to greater tumor invasiveness and a poor prognosis.
- Dampening the activity of tumor killer cells. M2 macrophages also play a role on inhibition of NK cell function through secretion of inhibitory factors and through cell-cell contact (78). By secreting PGE2, M2-like macrophages suppress NK cell cytotoxicity through downregulation of NKp30, NKp44, NKp46, and NKG2D by binding to E-prostanoid 2 (EP2) and EP4 receptors, leading to immunosuppressive cyclic adenosine monophosphate (cAMP)-protein kinase A (PKA) signaling in NK cells. PGE2 has also a direct impact on macrophages that we will discuss later. MMPs secreted by M2-like macrophages cleave FASL expressed at the NK cell surface thus inhibiting tumor cell apoptosis mediated by FAS : FASL ligation, which plays a critical role in immunosuppression. In addition, M2-like TAMs produce large amounts of IL-10, which inhibits both the production of IL-12 by intratumoral dendritic cells (DCs) and CD8+ T cell-mediated anti-tumor responses (79). IL-10, like TGF-β, suppresses CD8+ T cell and NK cell cytotoxicity by direct transcriptional repression of genes encoding functional mediators, such as perforins, granzymes, and cytotoxins. TGF-β contributes to metastasis allowing epithelial-to-mesenchymal transition of cancer cells, which empowers them with more invasive potential related to metastasis formation (80). TGF-β is associated with poor prognosis in multiple tumor types and has been demonstrated to be a major contributor of CD8+ T cell exclusion from tumors (81). M2 macrophages can also suppress T cell activation through depletion of tryptophan du to elevated expression of indoleamine 2,3-dioxygenase (IDO) and local depletion of L-arginine through Arginase1 (ARG1) expression (82).
- Promoting resistance to therapy. The ability of TAMs to limit the efficacy of immune checkpoint blockade therapy has been reported in several studies (83). Indeed, M2-like TAMs express high levels of both PD-1 and PD-L1, thus directly participating to immune suppression, as well as high levels of FcγRs that can quench therapeutic antibodies used for immune checkpoint blocking (84). In addition, as reviewed by C. Anfray (2019) (84) and A.Mantovani (2015) (85), TAMs seem to inhibit most anti-tumor treatments frequently used in clinical practice, including conventional chemotherapy, anti-angiogenic therapy, and radiation.
- Hence, high proportion of M2-like macrophages in TAMs might be a causative factor for poor prognosis and shorter survival of patients in several cancers such as chronic lymphocytic leukemia (86), T cell leukemia/lymphoma (60), oral cancer, thyroid cancer, bladder cancer (87) non-small-cell lung cancer (88) gastric cancer (50, 88, 89), clear cell renal cell carcinoma (89), ovarian cancer (46, 50, 88), breast cancer (7, 88) CRC (90–92) and laryngeal cancer patients (93). Moreover, in CRC patients, an increase in the proportion of M2 type TAMs was associated with an increase in liver metastases (94).

*In summary, M1-like TAMs that promote inflammatory responses against tumor cells mostly have an anticancer impact, whereas M2-like TAMs have anti-inflammatory activity that provides them with a pro-tumoral effect* (25).

### Macrophage receptors and signaling pathways that regulate macrophage polarization in cancer

#### Receptor signaling pathways leading to pro-inflammatory M1-like macrophages

Knowing that the TME plays a major role in macrophage polarization and that the interaction of chemokine/cytokine receptors with their ligands leads to the polarization of macrophages, we will describe in the next section the main signaling pathways involved in M1-macrophage polarization in humans through the different ligand-receptor interaction.

##### IFNγ receptors

**Figure 1:**
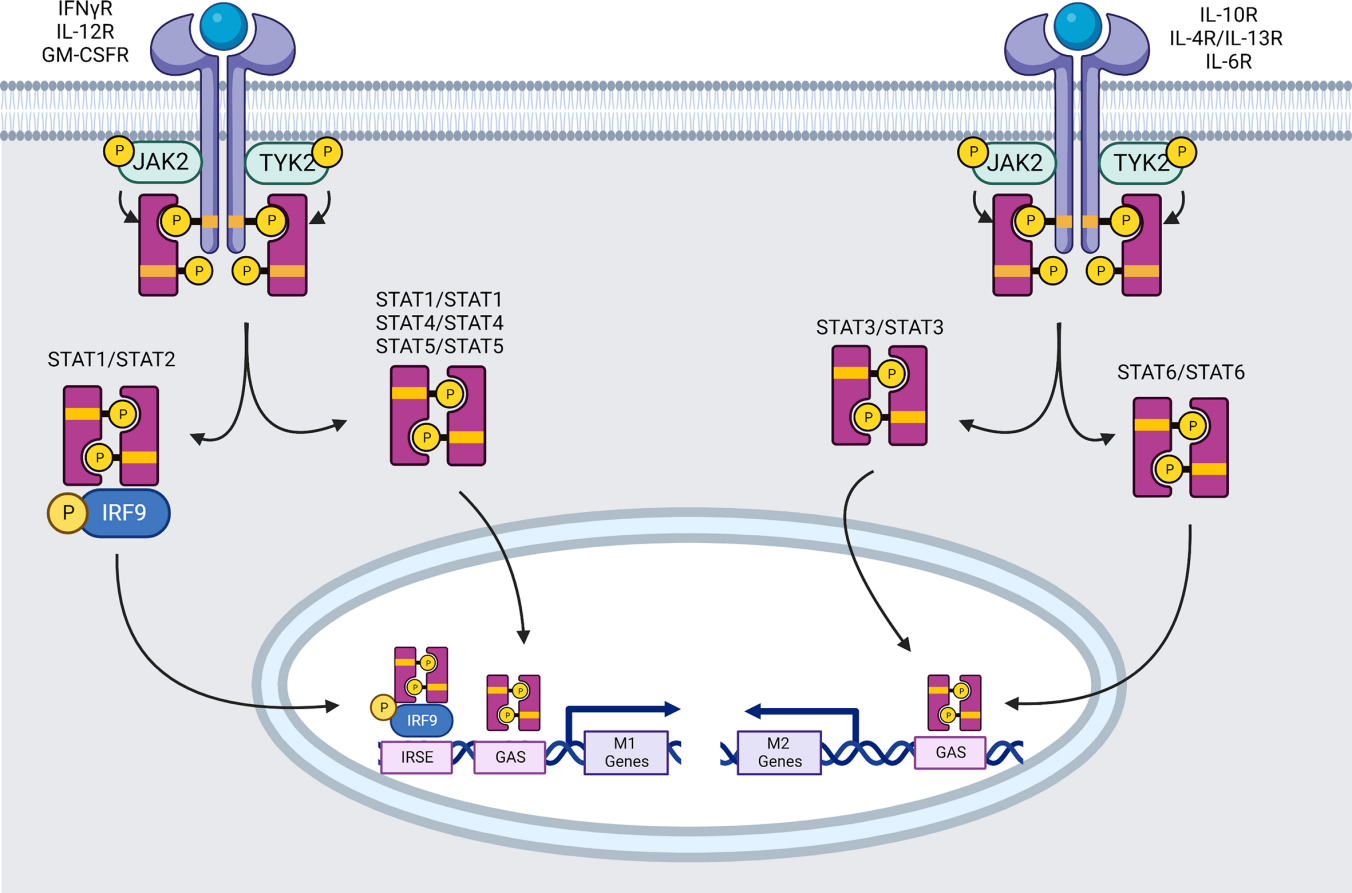
Signaling pathways mediated by the JAK family of tyrosine kinases (created using BioRender®).

IFNγR1 and IFNγR2 chains are members of the class II cytokine receptor family. Two chains of the IFNγR1 will first bind to an IFN-γ homodimer inducing subsequent recruitment of the two IFNγR2 chains. Both IFNγR chains must connect with a signaling machinery to transduce their signals. The intracellular domain of IFNγR1 has binding sites for Janus Tyrosine Kinase (JAK)1 and the latent cytosolic factor, signal transducer and activator of transcription (STAT)1. The JAK1- and STAT1-binding motifs are required for receptor phosphorylation, signal transduction, and induction of biological response (**Figure 1**). In the same way, the intracellular region of IFNγR2 contains a binding motif for recruitment of JAK2 kinase or Tyrosine Kinase 2 (TYK2) depending on ligand type for participation in signal transduction.

Within 1 minute of pro-inflammatory IFN-γ-therapy, the four essential downstream tyrosine kinases (JAK1, JAK2, TYK2, and STAT1) are phosphorylated (95). Ligand interaction leads to auto-phosphorylation and activation of JAK2, allowing JAK2 to transphosphorylate JAK1 (96). Activated JAK1 phosphorylates functionally important Y440 residue of each IFNγR1 chain, forming two contiguous docking sites for latent STAT1’s SRC homology 2 (SH2) domains (97). STAT1 pair is phosphorylated near the C-terminus at Y701, most likely by JAK2. Phosphorylation causes a STAT1 homodimer to dissociate from the receptor (98). The dissociated STAT1 homodimer reaches the nucleus and attaches to promoter regions, allowing or inhibiting transcription of IFN-regulated genes. The mechanism of STAT1 translocation into the nucleus remains unknown, however the participation of importin-1 (NPI-1) is suggested (99). STAT1 homodimers bind DNA at GAS sites in the TTCN (24, 25, 52) GAA consensus sequence (100) leading to expression of several genes. Two to five hundred genes are activated in response to the IFNs, including iNOS, MIG (=CXCL9), intercellular adhesion molecule 1 (ICAM-1), IRF1 and STAT1 himself (101). For example, ICAM-1 is known to inhibit M2 macrophage polarization (102), and its expression appears to interfere with M2-related phosphoinositide 3-kinase (PI3K)/Ak strain transforming (AKT) pathway reducing phosphorylation and expression of phosphatase and tensin homolog (PTEN) and AKT (103). iNOS and IRF1 are highly associated with inflammation and preferentially expressed in M1 phenotype (104).

STAT1 is also able to form heterodimers with STAT2 when type I and III IFN (α and λ) bind to IFNαR1:IFNαR2 or IFNλR1:IL-10R2 respectively. STAT2 is recruited following its activation on the tyrosine 466 (Y466) of IFNαR1, which has already been phosphorylated by TYK2. STAT2 then recruits STAT1, which can only be activated by phosphorylation on Y701 (105). STAT1-STAT2 heterodimers will join forces with IRF9 to create the Interferon-stimulated gene factor 3 (ISGF3) transcription complex. ISGF3 recognizes the IFN stimulated response elements (ISRE) (consensus region: YAGTTTC(A/T)YTTTYCC) promoter elements of pro-inflammatory macrophage specific genes induced by IFNs such as hypoxia-inducible factors (HIF)-1α (101, 106).

STAT1 phosphorylation at Serine (S)727 is also required for maximum transcriptional activation of target genes. STAT1 serine phosphorylation is induced by a variety of stimuli, including types I and II IFN, LPS, IL-2, IL-12, TNF-α, and platelet-derived growth factor. In macrophages, for example, LPS signaling enhances STAT1 S727 phosphorylation independently of Y701 phosphorylation, enhancing cellular responses to IFN-γ (107) thus contributing to polarization into M1 macrophages.

##### Il-12R

IL-12 has been mainly described for activating naïve Lymphocytes and inducing their differentiation. However, IL-12 also promotes M1 macrophage polarization by inducing IFN-γ production by Th1 cells, which in turn boosts anti-tumor cytotoxic CD8+ T and NK cells (108). IL-12p40 and IL-12p35 bind to IL-12R-β1 and -β2 respectively, resulting in transphosphorylation of associated JAKs (JAK2 and TYK2) and then lead to the activation and translocation of STAT4 homodimer into the nucleus where they bind to STAT binding sites in the IFN-γ promoter of targeted M1 genes (109, 110).

Furthermore, IL-12 acts as a M1 classical activating factor through an indirect mechanism. Indeed, IL-12 promotes antigen presentation by increasing the expression of MHC-I on tumor cells (111), promoting polarization to M1 macrophages, and recruiting effector immune cells by increasing the expression of the chemokines CXCL9, CXCL10, and CXCL11 (112).

IL-12 has been also studied in pathological context showing again its ability to favor M1 polarization. In fact, it has been shown that IL-12R/STAT4 drives obesity-associated insulin resistance through pro-inflammatory M1 macrophage polarization (113).

##### Toll like receptors and IL-1R

The evolutionarily conserved Toll-like receptors (TLRs) family is one of the most studied families of pattern recognition receptor (PRRs) and plays a crucial role in early host defense against invading pathogens (40).

Among the 13 members of the TLR family including transmembrane TLR1, TLR2, TLR4, TLR5, TLR6, and TLR10, and endosomal TLR3, TLR7, TLR8, TLR9, TLR11, TLR12, and TLR13, the most relevant TLRs related to macrophage polarization in cancer context are TLR2/TLR6 (114), TLR3 (114, 115), TLR4 (115, 116) and TLR7/8 (117). TLR4, for example, activates signaling cascades (NF-κB and MAPK) that result in production of proinflammatory cytokines (TNFα, IL-1, IL-6, IL-12) and type-I interferons, which are essential for the propagation of the inflammatory response.

**Figure 2:**
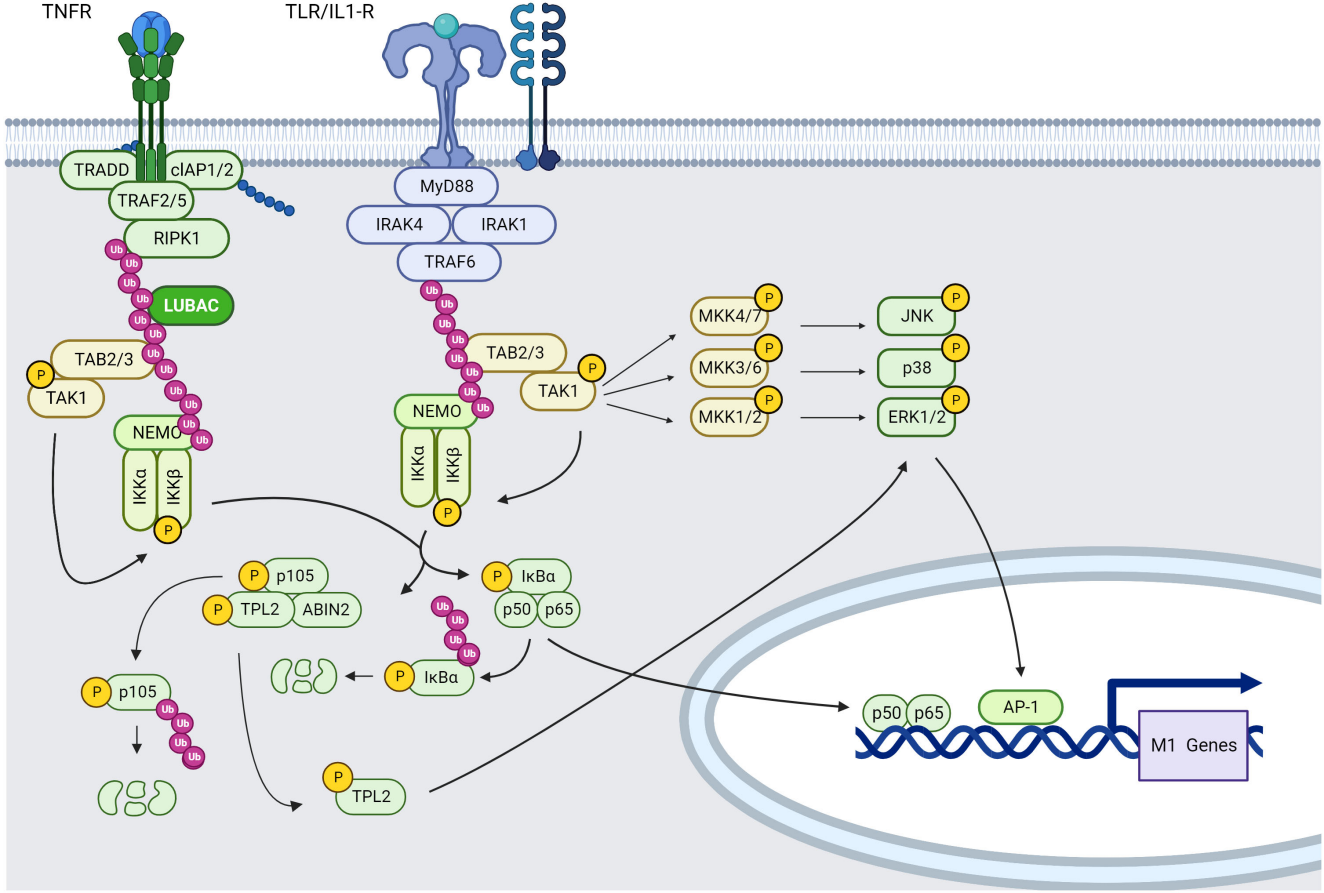
TNFR and TLR/IL-1R, NF-κB and MAPK signaling pathways in macrophages (created using BioRender®).

TLRs are type I integral membrane glycoproteins that belong to a wider superfamily that includes the IL-1 receptors, due to significant similarities in their cytoplasmic region known as the Toll/IL-1R (TIR) domain. TLRs/IL-1Rs dimerize following ligand binding and undergo the conformational shift necessary for the recruitment of pro-inflammatory downstream signaling molecules. These include the adaptor molecule myeloid differentiation primary-response protein 88 (MyD88), IL-1R-associated kinases (IRAKs), TGF-activated kinase (TAK1), TAK1-binding protein 1 (TAB1), TAB2, and TNFR-receptor-associated factor 6 (TRAF6) (118) (**Figure 2**).

TLR4, TLR2, and TLR2/TLR6 recruit MyD88 *via* the bridging adaptor MAL, whereas TLR7/TLR8 recruit MyD88 directly. TIR-domain-containing adapter-inducing interferon-β (TRIF) is also recruited indirectly to TLR4 *via* the bridging adaptor TRIF-related adaptor molecule (TRAM), whereas TLR3 recruits TRIF directly. TLR4 is the only receptor that uses TRIF, TRAM, MyD88, and MyD88 adaptor-like protein (MAL/TIRAP). As a result, it serves as a model for both the TRIF- and MyD88-dependent pathways (119).

- MYD88-dependent TLR signaling pathway
  MyD88 is recruited to the cytoplasmic TIR domain, where it enhances IRAK4-receptor complex attachment *via* a homophilic Death Domain (DD) interaction. MyD88 binding to IRAK4 enhances IRAK4-mediated phosphorylation of S376 and T387 residues of IRAK1 and subsequent autophosphorylation, resulting in IRAK1 kinase activity (120, 121).
  IRAK1 interacts then with TRAF6. TRAF6, in collaboration with the ubiquitin-conjugating enzymes UBC13 and UEV1A, promotes K63-linked polyubiquitination of several target proteins including TRAF6 itself, Inhibitor of kB kinase gamma (IKKγ/NEMO) and the TAK1 protein kinase complex. Regarding TAK1, TRAF2/5 (through TNF-α stimulation) or TRAF6-mediated K63 ubiquitination leads to recruitment of TAK1-binding protein (TAB)2 or TAB3 in order to form the TAK1 protein kinase complex composed of TAK1, TAB2 or TAB3, which phosphorylates and activates the inhibitory kappa B kinase alpha/beta (IKKα:IKKβ)–NF-κB complex and MAPK kinases (122–124), resulting in the expression of M1 type pro-inflammatory cytokines. Moreover, IKK, MAPK signaling and cytokines in TAK1-deficient murine peritoneal macrophages stimulated with LPS are slightly reduced compared with wild-type controls (122). More precisely, the TAB2:TAB3:TAK1 complex will phosphorylate mitogen-activated protein kinase Kik 4 (MKK4) and MMK7 leading to c-Jun N-terminal kinases (JNKs) phosphorylation, thus contributing to the acquisition of a M1 macrophage phenotype. Moreover, TAK1 can phosphorylate MKK3 and MKK6 leading to P38α phosphorylation and then MK2 phosphorylation resulting into pro-inflammatory cytokine production such as IL-1β and TNF-α. TAK1 can be also activated by many stimuli, including IL-1β, TNF-α, TLR ligands, and B and T cell receptor ligation (125).
  MAP/ERK kinase (MEK)/extracellular signal-regulated kinase (ERK) pathway activation occurs preferentially *via* the activation of the MAP3K tumor progression locus 2 (TPL2; also known as MAP3K8) (126). Indeed, TPL2 is activated *via* IκB kinase (IKK)-induced proteolysis of the NF-κB subunit precursor protein p105, IKKβ phosphorylation of p105 causes ubiquitination and partial degradation of p105, resulting in the release of TPL2. Free TPL2 will phosphorylate MKK1 and MKK2 (also called respectively MEK1 and MEK2) and subsequently activates ERK signaling pathway. IKKβ also directly phosphorylates TPL2 on S400, which is essential for LPS-mediated pro-inflammatory activation of macrophages *via* ERK (127).
  These observations show a direct link between MAPK activation and NF-κB activation in IL-1R and TLR-stimulated macrophages. Moreover, MAPK and IKK signaling in macrophages plays a pro-inflammatory role, suggesting that modulation of NF-κB, JNK, ERK and P38 pathways could constitute a potential therapeutic target regulating tumor homeostasis and favoring immunostimulatory context.
- TRIF-dependent TLR signaling pathways

TRIF-dependent signaling leads to Type-I IFNs production. TRIF can recruit both TRAF3 or TRAF6, TRAF3 recruits then the IKK-related kinases TBK1 and IKKε, as well as NEMO, to phosphorylate IRF3. IRF3 then forms a dimer and translocate from the cytoplasm into the nucleus, where it stimulates the transcription of type I IFN genes (128).The recruitment of TRAF6 activates the TAK1 complex leading to downstream signaling pathways previously described.

It has been recently shown that STAT3 acts directly on TLR4 signaling pathways *via* interaction with TRAF6 and TBK-1, resulting in non-canonical STAT3 S727 phosphorylation but not Y705, thus inducing metabolic M1 reprogramming and inflammation (129).

In the TME, cellular components released during necrosis act as DAMPs, that can also be ligands for TLRs and assist the establishment of a pre-programmed pro-inflammatory M1 or “classically activated” phenotype. This is accomplished *via* enhanced activation of signaling pathways including NF-κB, P38 MAPK, and others, which govern the production of pro-inflammatory cytokines (e.g., IL-1, IL-6, and IL-12) (130–132).

Meanwhile, stimulation of TLR3 and TLR4 may also activate a MyD88-independent pathway that induces IFN-β secretion. This pathway is mediated by Toll/IL-1 receptor (TIR) domain-containing adaptor and knockdown of TIR led to blockade of TLR4 mediated inflammation (119, 133, 134).

*All ligands for each TLR and related functions are detailed in S. Akira and K. Takeda review* (135).

##### Tnfr

TNF receptor superfamily TNFRSF is made up of 27 physically similar receptors that bind one or more molecules from the TNF superfamily (TNFSF), composed of more than 20 structurally related proteins (ligands). TNFSF ligands are membrane-anchored or soluble trimers that cluster their cognate cell surface receptors to start signal transduction. TNFSF ligands and receptors have distinct structural properties that relate them to cell growth, survival, or death, while some molecules can activate both inflammatory and cell death pathways, depending on target cell types and other external stimuli. Many of the TNFRSF molecules, such as CD40, TNFR1 and TNFR2 for example, are expressed in immune cells, indicating that they may have a role in autoimmune and inflammatory illnesses, as well as in cancer.

Moreover, TNFα is a positive regulator of M1 polarization *via* the NF-κB pathway. Therefore, among the superfamily of TNFR, we will describe more precisely TNFR1 and TNFR2 that are TNFα receptor subunits and are highly expressed in TAMs (136).

TNFR1 and 2 are single-spanning type I transmembrane proteins characterized by several cysteine-rich domains (CRDs) in their extracellular domain that are not directly involved in ligand binding but mediate inactive self-association in the absence of ligand. Regarding the cytoplasmic moiety, TNFR1 harbors a DD allowing protein-protein interaction with cytoplasmic proteins also harboring a DD domain (137).

After binding TNF-α, TNFR1-associated death domain (TRADD) and receptor-interacting serine/threonine-protein kinase 1 (RIPK1) are recruited by the TNFR1 *via* DD-DD interactions inducing the recruitment of TRAF2 homotrimers and E3 ligase, leading to NF-κB classical signaling. Indeed, TRAF2:TRAF2 homodimers (or TRAF3:TRAF3) already form complexes in the cytoplasm with the E3 ligases cIAP1 and cIAP2 and are recruited on TRADD. RIPK1 will be modified by addition of K63-linked ubiquitin chains that will serve as docking site for the LUBAC complex. This complex will ubiquitinate NEMO, a member of the IKK complex that interacts with TRAF2 *via* its IKK2 subunit, the TAK1-TAB2 complex interacts with K63-ubiquitin modified RIP1 *via* the TAB2 K63-ubiquitin binding subunit. Activated TAK1 then phosphorylates IKK2 leading to phosphorylation of iκBα and its subsequential K48-ubiquitination and degradation. This degradation will allow p50/p65 NF-κB dimer translocation to the nucleus inducing pro-inflammatory gene expression (138) (**Figure 2**).

TNFR1 signaling complex starts internalizing and this comes along with the release of the TNFR1-bound signaling molecules that trigger apoptosis through caspase-8 or necroptosis through RIPK3:RIPK1 complex formation. Apoptotic cells produce membrane-enclosed apoptotic vesicles carrying the dying cell’s material, which are removed by macrophages during the resolution of inflammation. Necroptosis, on the other hand, is a lytic form of cell death that causes inflammation by releasing intracellular DAMPs and proinflammatory cytokines (139).

TNFR2 engagement also results in activation of the classical NF-κB pathway and to the recruitment of TRAF2-cellular inhibitor of apoptosis proteins 1 and 2 (cIAP1/2) and TRAF1-TRAF2-cIAP1/2 complexes. This phenomenon can lead to a significant depletion of these complexes in the cytoplasm and may affect other activities of these molecules inducing activation of the alternative NF-κB pathway through TRAF3: MAP3K NF-κB-inducing kinase (NIK) complex formation (140).

cIAP1/2 are also implicated into alternative NF-κB pathways through degradation of NIK which activates IKKα, then phosphorylates p100 on NF-κB2 (p52/p100), leading to p100 proteasomal degradation and release of p52. After binding to RelB, p52 is translocated to the nucleus to control gene transcription (139).

TNFR1 also triggers the MAPK cascades leading to the activation of ERK, JNK (141) and P38 (142). TNFR1 activates a MAP3K called apoptosis-signaling kinase-1 (ASK-1) that associates with TRAF2 in the TRADD-RIPK1-TRAF2 complex, activating MAP2Ks, JNK/ERK kinase-1 (SEK1, also known as MAPK-kinase (MAP2K)-4), and MAP2K-7, which in turn activates JNKs, MAP2K-3 and MAP2K-6 which activate P38 MAPK (143). TNFRs also activate the ERK1/2 signaling pathway *via* activation of TPL2-MAP2K1/2 (also called TPL2-MEK1/2-ERK) pathway through activation of NF-κB pathway as described before (144). It has also been documented that TRAF2 initiates P38 activation by binding two proximal protein kinases: GCK and RIP. GCK and RIP, in turn, signals by binding MAP3Ks upstream of the JNKs and P38s. The signaling cascade downstream of TRAF2 is well known to regulate and mediate proinflammatory responses leading to macrophage production of cytokines and type I IFN (145).

Another interesting receptor from TNFR family in terms of macrophage polarization is the trans-membrane costimulatory receptor CD40. CD40 is expressed on macrophages and was shown to play crucial roles in autoimmune and infectious diseases, transplant rejection and tumor regression. CD40 can transduce signals that regulate a wide range of cellular responses, from proliferation and differentiation to growth repression and cell death. The ligand of CD40, CD154 (=CD40L or GP39), has a bidirectional effect on antigen-presenting cells. Indeed, CD40-CD154 interaction activates ERK1/2 leading to synthesis of anti-inflammatory IL-10 and phosphorylation of P38 that results in pro-inflammatory IL-12 production depending on signaling strength. It has also been shown that CD40-CD154 interactions can activate macrophages and is required for production of NO (146).

CD40 triggers TRAF6 pathway leading to SRC/MEK/ERK signaling (147) previously described and can activate others MAPKs. As the other members of TNFR family, CD40 can trigger NF-κB pathway through TRAF6 (148). Transduction downstream of CD40 also involves the JAK3/STAT3 and PI3K/AKT pathway activation (149) known as pro M2-type signaling pathways.

All these observations show that TNFR family might be a major player in M1 to M2 macrophage balance in the TME.

##### Gm-csfr

GM-CSF interaction with its receptor (GM-CSFR) triggers M1 polarization of macrophages, with the generation of proinflammatory mediators such as TNF-α, IL-6, IL-12, and IL-23 (150).

**Figure 3:**
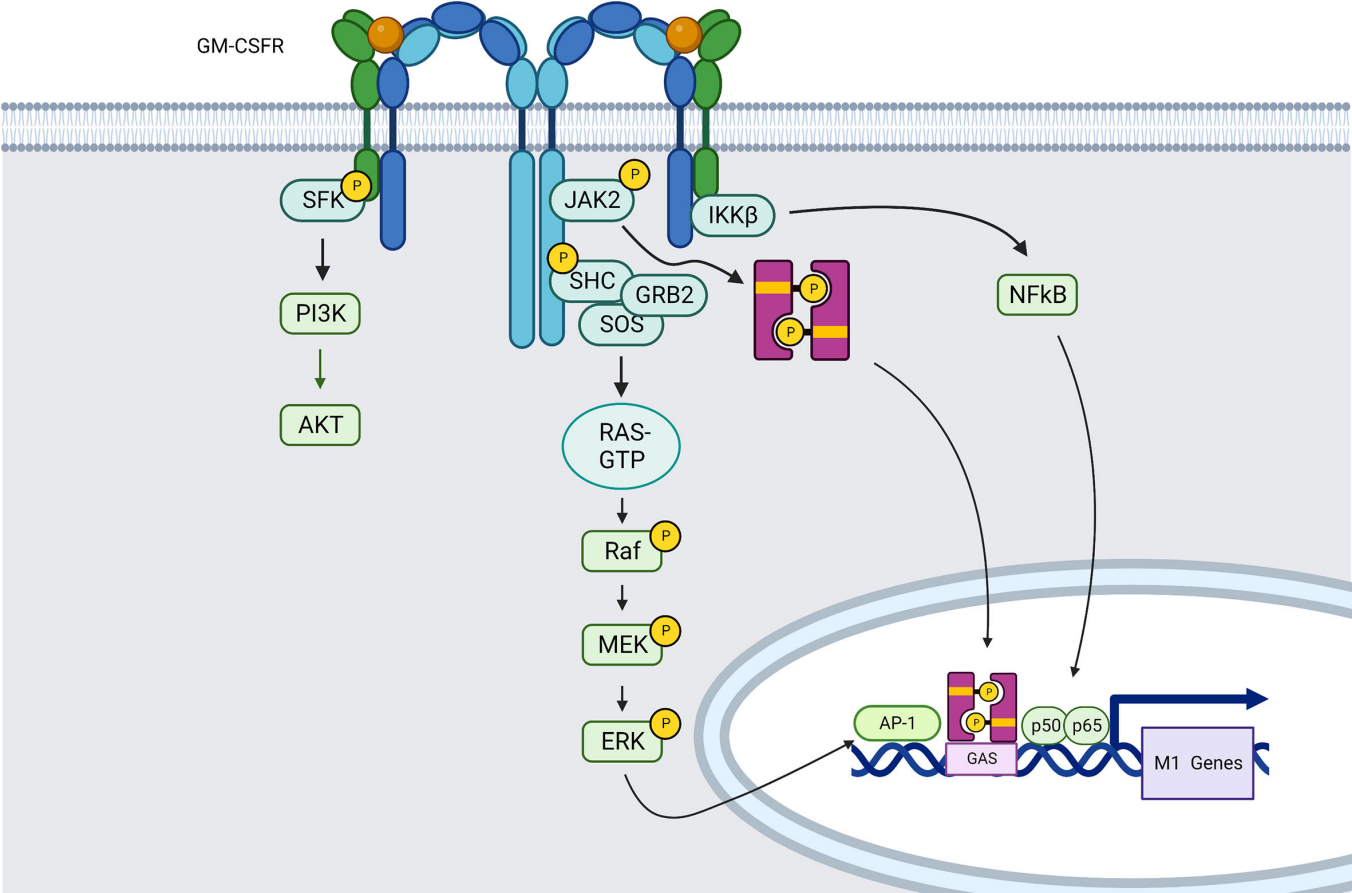
GM-CSFR induced signaling pathways in macrophages (created using BioRender®).

GM-CSF can interact with the two receptor subunits of GM-CSFR. The first component is the ligand-specific α-chain (GM-CSFRα), which forms a complex with the b-chain (GM-CSFRβ) to form GMC-SFR. The β-chain is also shared by IL-3 and IL-5. These complexes of GM-CSF–GM-CSFRα–GM-CSFRβ create first hexameric and then dodecameric ligand–receptor complexes, which will trigger JAK/STAT pathway and especially the JAK2–STAT5 (151) pathway that favor pro-inflammatory polarization of macrophages (152), but also NF-κB, PI3K/AKT, and growth factor receptor-bound protein 2 (GRB2)/MEK/ERK signaling pathways (151, 153) (**Figure 3**), which will be described later in this review.

Indeed, MAPK pathways can also be directly activated by receptor tyrosine kinases (RTKs) such as GM-CSFR, among others. Ligand-induced receptor dimerization increases receptor activation and Tyrosine autophosphorylation in the intracellular region. In the case of MEK/ERK pathway for example, the phosphorylated residues serve as binding sites for proteins like GRB2 that have SH2 or phosphotyrosine-binding (PTB) domains. Son of sevenless (SOS), a guanine nucleotide exchange factor (GEF) is recruited from the cytosol to the plasma membrane by GRB2, where it drives the exchange of guanosine diphosphate (GDP) bound to RAS by guanosine triphosphate (GTP), which is necessary for positive control of RAS activity. RAS may interact directly with its target effectors, one of which is RAF, thanks to this nucleotide exchange. Activated RAF binds to and phosphorylates the dual-specificity kinases MEK1/2, which then phosphorylate ERK1/2 in their activation loop *via* a conserved Thr-Glu-Tyr (TEY) motif (121) (**Figure 3**).

##### NOTCH receptor

The Notch signaling pathway is widely acknowledged to have a critical role in controlling development and assisting in the regulation of the response to various stimuli. Hitherto, four NOTCH receptors and five NOTCH ligands have been identified. NOTCH receptors attach to members of their ligand families, Delta-like proteins (DLLs) and Jagged (JAG) proteins, inducing the release of NOTCH intracellular domain (NICD) into the cell nucleus, then binding to recombination signal binding protein for immunoglobulin kappa J (RBP-J) to form a transcriptional IRF8 complex, thus promoting target M1 gene expression (154). In a non-canonical way, Notch can also activate mechanistic target of rapamycin (mTORC2)/AKT and NF-κB through its NICD (155).

NOTCH1 expression is increased in M1 macrophages, and its inhibition was shown to enhance M2 polarization (156). Furthermore, in a mouse model of breast cancer (mouse mammary tumor virus-polyoma middle tumor-antigen (MMTV-PyMT)), hyperactivation of Notch signaling, particularly in TAMs, was found to inhibit tumor development (157).

Moreover, in the literature both M1 and M2 phenotypes could be generated in TAMs through Notch pathway depending on the upstream ligand-Receptor involved. For example, *in vitro* coincubation of macrophages with cells expressing DLL4 can lead to activation of NOTCH1 signaling and result in pro-inflammatory genes expression, such as IL-12 and iNOS (158, 159). Moreover, the blockade of DLL4 using an antibody reduced pro-inflammatory macrophage accumulation in inflammatory lesions and attenuated atherosclerosis and metabolic disease, while NOTCH1/JAG1 signaling was shown to regulate anti-inflammatory macrophage activation and IL-10-producing TAMs (160).

Research about how Notch signaling is controlled in TAMs and translated into pro- or anti-tumor actions is still in its early stages (158).

##### Trem-1

Triggering receptor expressed on myeloid cells 1 (TREM-1) is found mostly on myeloid cells such as monocytes/macrophages and granulocytes. TREM-1 exists in two forms: as a membrane-bound receptor and/or as a soluble protein. In its membrane bound form, TREM1 is composed by three different domains: an immunoglobulin-like domain that is responsible for ligand binding, a transmembrane portion, and a cytoplasmic tail that interacts with the immunoreceptor tyrosine-based activation motif (ITAM) of adaptor molecule DAP12. This interaction leads to DAP12 tyrosine phosphorylation and results in downstream signal transduction through ζ-associated protein of 70 kD (ZAP70) and spleen tyrosine kinase (SYK) recruitment. SYK then recruits and tyrosine phosphorylates adaptor complexes including Casitas B-lineage Lymphoma (CBL), SOS and GRB2, resulting in downstream signal transduction through the PI3K, phospholipase-C-gamma (PLC-γ), and ERK pathways. These pathways result in transcription factor activation, including ETS domain-containing protein (Elk1), nuclear factor of activated T-cells (NFAT), AP-1 (c-Fos and c-Jun), and NF-κB, which trigger transcription of genes encoding pro-inflammatory cytokines such IL-1β, chemokines such as CCL2, and cell-surface molecules such as CD86 and MHC class II (161, 162).

#### Receptor signaling pathways leading to anti-inflammatory M2-like macrophages

As for M1 macrophages, the interaction of chemokine/cytokine receptors with their ligands present in the TME is the main actor of M2 macrophage polarization through receptor signal transduction and downstream signaling pathways. Therefore, we will describe in the next part, these main mechanisms implicated in M2 polarization in a tumor context.

##### Mcs-r

Also known as CSF1R, this receptor binds M-CSF also named CSF-1. MCSF-R is also activated upon IL-34 engagement, thus representing the only RTK known to be activated by two ligands of unrelated sequence. MCSF and IL-34 support the expression of CD11b and of chemokines, cytokines, and other plasma membrane markers characteristic of M2 polarization (163).

**Figure 4:**
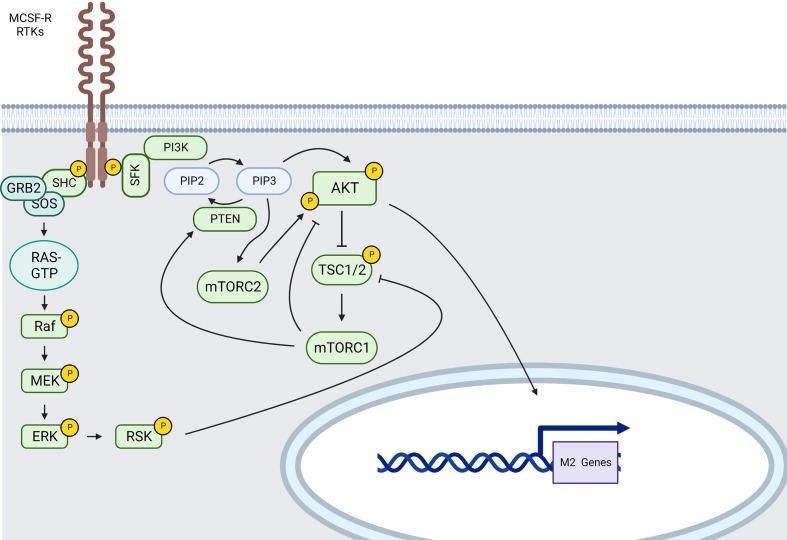
MCSF-R and RTKs main signaling pathways involved in M2-like polarization (created using BioRender® ).

M-CSF binding to MCSF-R causes fast dimerization of the receptor, a first wave of tyrosine phosphorylation of MCSF-R, and the creation of complexes between the CSF-1R and SFK, CBL, the regulatory subunit of PI3K (p85) and p110δ, GRB2, and other signaling molecules, many of which get tyrosine-phosphorylated (**Figure 4**).

Indeed, after engagement of the MCSF-R, a cascade of downstream signaling molecules, including those involved in the PI3K/AKT and MAPKs signaling pathways (164), is activated, boosting survival and differentiation of M2-like macrophages.

Recruitment of PI3K will then induce phosphorylation of phosphatidylinositol-4, 5-bisphosphate (PIP2) to catalyze phosphatidylinositol-3, 4, 5-triphosphate (PIP3) at the plasma membrane; PIP3 further recruits AKT through is pleckstrin homology domain (PH-domain) causing a conformational change and the phosphorylation of AKT at T308 by 3-Phosphoinositide-dependent protein kinase 1 (PDK1) (165). PIP3 also recruits the mTORC2 complex and enhances AKT activation by mTORC2 *via* S473 phosphorylation. When AKT is activated through its phosphorylation, it subsequently phosphorylates and inactivates the tuberous sclerosis complex (TSC) 1/2. TSC1/2 inhibition by AKT activates mTORC1 *via* Ras homolog enriched in brain (Rheb) suppression (166). mTORC1, such as JNK1 (167), can then induce PTEN and subsequent AKT pathway inhibition. AKT inhibition abrogates the upregulation of several M2 genes (168). Nevertheless, individual AKT2 isoforms also contribute to M1 polarization (169). Indeed, AKT is a serine-threonine protein kinase family composed of three isoforms expressed from independent genes (AKT1/PKBα, AKT2/PKBβ and AKT3/PKBγ) (170) and there is data suggesting that the outcome of macrophage polarization upon AKT activation depends on the AKT isoform involved. Indeed, AKT1 ablation results in M1 polarization whereas AKT2 ablation results in M2 polarization (169). Reciprocally, AKT2-deficient macrophages present with enhanced IL-10 secretion upon LPS stimulation (171).

AKT isoform-specific effects on macrophage function have been also reported for PI3K and isoform-specific effects on macrophage function have been reported both for AKT and PI3K (166, 170, 172, 173). Indeed, PI3K p110δ, p110β, p110γ isoforms seem to be associated with M2 macrophage polarization whereas p110α seems to lead to M1 macrophage polarization (170). The most characterized and expressed isoform in myeloid cells is p110γ. In fact, it has been shown that P110γ/AKT/mTOR-mediated immune suppression promotes tumor growth and that P110γ inhibition suppressed myeloid cell adhesion and recruitment into tumors. p110γ inhibition also reverses tumor growth and mediated immune suppression by inducing proinflammatory gene expression in TAMs through NF-κB inhibition (174, 175). However, more data would be needed to accurately assess the roles of the other different PI3K isoforms in macrophage polarization.

Other important actors implicated into PI3K/AKT/mTOR pathway have been identified. For example, the lipid phosphatase PTEN is a negative regulator preventing overactivation of the AKT/mTOR pathway. Mice treated with a myeloid-specific PTEN deletion present a larger number of M2 macrophages releasing less TNF-α and more IL-10 in response to TLR ligands (176). AMPK is an inhibitor of mTOR activity resulting in a stronger reduction of the anti-inflammatory cytokine production (IL-10) as well (177).

During macrophage response to M-CSF, ROS promotes AKT1, P38 and JNK activation (178, 179). PI3K regulates ERK phophorylation in macrophages treated with M-CSF resulting in VEGF production (180, 181). Internalized MCSF-R induces by CBL ubiquitination mediates sustained ERK1/2 and AKT signaling (182). GRB2 direct recruitment is also involved in MCSF-R-mediated transient ERK activation when it is associated with the GEF, SOS (183). ERK can activate ribosomal S6 kinase (RSK) that inhibits TSC1/2 leading to mTORC1 activation. These observations show that MAPKs and more particularly ERK1/2 activation can be linked to both M1 and M2 polarization of macrophages.

Among the several signaling cascades described as downstream of MCSF-R, PI3K/AKT is the most relevant and its downstream targets are critical in M2 macrophage polarization. This pathway is also important in limiting pro-inflammatory responses and increasing anti-inflammatory responses in TLR-stimulated macrophages, and was identified as a negative regulator of TLR and NF-κB signaling in macrophages (184).

*All MCSF-R intracellular phosphorylation sites and related functions are detailed in E. Richard Stanley and Se Hwan Mun review* (185).

##### Interleukin Receptors

###### Il-10R

IL-10 receptor is composed of at least two subunits, IL-10Rα and IL-10Rβ, which are members of the interferon receptor (IFNR) family. IL-10R signaling is mainly relayed by the JAK/STAT system (**Figure 1**). Indeed, IL-10Rα is constitutively associated to JAK1, whereas IL-10Rβ is constitutively associated to TYK2 (186). Upon IL-10 engagement, these kinases phosphorylate transcription factor of the STAT family. STAT3 is a transcriptional regulator acting downstream of IL-10 signal, a crucial anti-inflammatory cytokine. IL-10 has also been proven to be a key mediator in the resolution of inflammation. Conditional genetic inactivation of STAT3 in mouse macrophages revealed the role of STAT3 in the regulation of inflammation since these animals presented with decreased bactericidal activity and increased production of pro-inflammatory cytokines IL-12, IL-6, TNF-α, IL-1β, IFN-γ in response to LPS, and were refractory to IL-10 treatment (187, 188). Myeloid cells exhibit robust STAT3 activation in response to recombinant IL-10 variants across a wide range of IL-10Rβ–binding affinities (188). Moreover, IL-10-mediated inhibition of IFN-induced gene transcription (CXCL10, ISG54, ICAM-1) in human monocytes correlates with inhibition of IFN-induced STAT1 activation and tyrosine phosphorylation (189). Levels of IL-6, IL-8, and TNF-α produced by primary human monocyte-derived macrophages stimulated with LPS were strongly decreased upon IL-10 treatment (188).

Furthermore, induction of IL-10 in macrophages by proinflammatory signals requires activation of AKT (190) and IL-10 also promotes M2 polarization through the induction of p50 NF-κB homodimer, c-Maf, and STAT3 activities (191). Finally, IL-10 inhibit LPS-Induced MAPK activation (192).

###### Il-4R/Il-13R

There are two types of IL-4 receptors, namely Type I receptors, which are composed by the IL-4 receptor α-chain (IL-4Rα) and the common gamma chain (γc), and type II receptors, which are composed by IL-4Rα and the IL-13 receptor α-chain 1 (IL-13Rα1). IL-4 initially binds to IL-4Rα with high affinity, which then recruits γc or IL-13Rα1 to create type I or type II ternary ligand-receptor complexes, respectively.

The activation of the receptor-associated JAKs results in the phosphorylation of tyrosine residues in the cytoplasmic domain of the IL-4Rα, which then serve as docking sites for the recruitment of other signaling molecules, including STAT6, the primary STAT protein activated in response to IL-4 stimulation. JAKs can tyrosine-phosphorylate STAT6, causing it to disengage from the receptor and dimerize *via* reciprocal contacts between its SH2 domain and the phosphotyrosine 641 (Y641) on another STAT6 molecule (193). STAT6 homodimers translocate to the nucleus, where they bind to specific DNA patterns inside the promoters of responsive target genes and begin M2 gene transcription (57) (**Figure 1**).

Alternatively, IL-4 can signal by recruiting insulin receptor substrate (IRS) proteins to specific phosphotyrosine residues on the IL-4Rα, where IRS (mainly IRS2) can be phosphorylated and recruit other signaling molecules such as the p85 subunit of PI3K. PI3K activation has been identified as a critical step in macrophage M2 activation in response to IL-4 and it has been shown that a crosstalk between the STAT6 and PI3K pathway is required for IL-4–induced M2 macrophage activation in SHIP-deficient macrophages (193). IRS recruitment of PI3K leads to the activation of the downstream protein serine/threonine kinase AKT/mTOR pathways. IRS can also interact with GRB2, which is complexed to SOS and causes Ras and the downstream MAPK pathway activation.

With the development of genomic technology during the last decade, data on gene expression patterns in IL-4-stimulated macrophages has gathered. Hao-Wei Wang and Johanna A Joyce have summarized IL-4-induced gene sets in TAMs in mouse and human in their review named Alternative activation of tumor-associated macrophages by IL-4 Priming for protumoral functions (193).

The characterization of these cellular interactions may lead to novel strategies for disarming TAMs’ tumor-promoting functions by targeting either upstream regulators (e.g., IL-4) or downstream effectors (e.g., cathepsins, EGF signaling), and could have potential as monotherapies or complements to conventional anticancer therapies.

###### Il-6R

IL-6 is a prominent proinflammatory cytokine released during infection or tissue injury that contributes to both innate and adaptive immune responses (194). It is worth noting that only a few cell types, namely macrophages, neutrophils, CD4+ T cells, podocytes and hepatocytes, express IL-6R on their cell surface and may thus respond directly to IL-6. In particular, it is known that IL-6 modulates monocyte differentiation towards macrophages and DCs (195).

IL-6 requires two distinct receptors to trigger signaling, IL-6R and gp130. IL-6 can attach to membrane IL-6R in a classic manner or to soluble IL-6R in a trans-signaling manner or be presented by T cells *via* DC expressing IL-6R. A hexamer complex with gp130 is produced in all three modalities of IL-6R signaling. The NF-κB and JAK/STAT3 pathways are activated by IL-6 binding and influence the polarization of macrophages to M2-Arg1 expressing macrophages (196) (**Figure 1**).

In cancer, TAMs were reported to secrete IL-6 *via* STAT3 pathway leading to expansion of cancer stem cells and breast cancer progression. Furthermore, IL-6 promotes M2 macrophage polarization, while concurrently stimulating TNBC stemness and tumor progression (197–199).

##### TGFβR

The transforming growth factor (TGF-β) superfamily includes 32 secreted proteins as well as three receptors. These proteins are implicated in the development of a variety of fibrotic diseases. Moreover, TGF-β is important for immune suppression in the TME, being involved in tumor immune evasion and poor response to cancer immunotherapy. In addition of being secreted by M2-like TAMs, TGF-β promotes M2-like phenotype and IL-10 secretion and decreases TNF-α and IL-12 cytokine secretion *via* SNAIL signal transduction (200).

**Figure 5:**
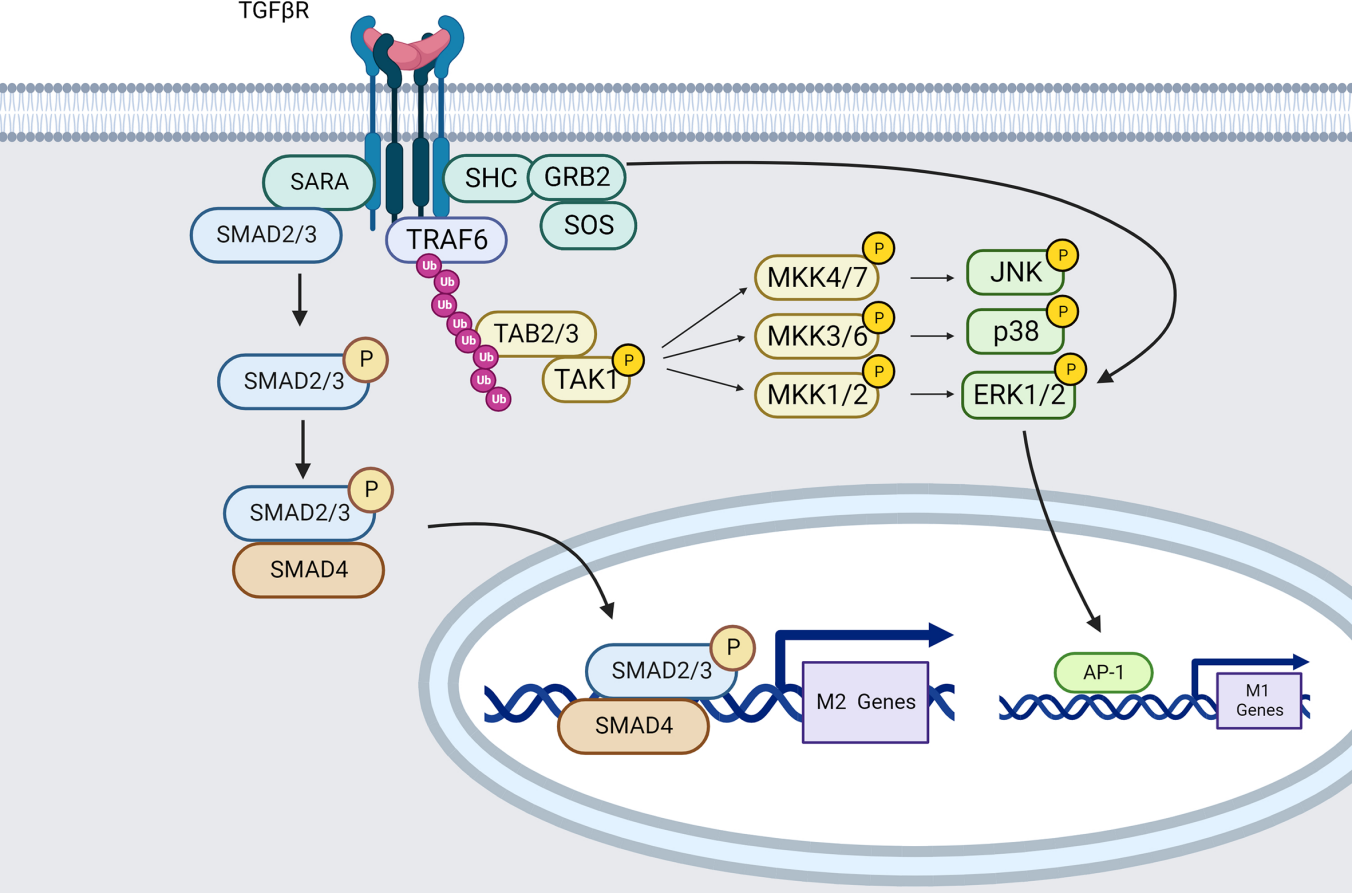
TGFβR signaling pathways in macrophages (created using BioRender® ).

TGF-β binds to three isoforms of the TGF-β receptor (TβR). TβRI and RII are both serine/threonine and tyrosine kinases, while TβRIII does not have any kinase activity. Phosphorylation of TβRI/RII is necessary for activating canonical or noncanonical signaling pathways, as well as for regulating the activation of other signaling pathways (201) (**Figure 5**).

The most extensively studied downstream mediators of TGF-β signaling are SMAD-dependent pathways. The TβRII subunit autophosphorylates and phosphorylates TβRI upon TGF-β engagement. As a result, the TβRI kinase domain interacts with the transcription factors SMAD2 and SMAD3 *via* the SMAD anchor for receptor activation (SARA) (202). TRβI then phosphorylates and activates SMAD2/3. When SMAD2/3 becomes active, it attaches to SMAD4, and the SMAD2/3/4 complex is translocated into the nucleus leading to ARG1 expression (203).

Data from Zhang et al. also demonstrate that blockade of the SMAD2/3 pathway reverses the immunophenotype of TGF-β induced macrophages from an anti-inflammatory M2-like phenotype to a pro-inflammatory M1 phenotype. TGF-β can also induce M2-macrophage polarization by up-regulating SNAIL expression through SMAD2/3 and PI3K/AKT signaling pathways (200).

In opposition, TGF-β signaling has also been characterized as using SMAD-independent pathways. Indeed, TRAF6 can bind TGF-β receptors to TAK1 leading to P38 MAPK, JNK and ERK-mediated M1 phenotype (204, 205).

##### Tie2

Angiopoietins (ANG)-1 and -2, as well as their receptor, the Tek tyrosine kinase receptor TIE2, are essential for controlling angiogenic processes throughout development, homeostasis, cancer, inflammation, and tissue repair. TIE2 is expressed by macrophages and has been proposed to contribute to solid tumor development by supporting immunosuppressive activities associated with M2 macrophage polarization.

TAMs expressing TIE2 have been named TIE2 expressing macrophages (TEM). TIE2 is a receptor for ANG1–4. In breast cancer and glioma, intratumoral TEMs are found near to nascent tumor vasculature and were found to have proangiogenic activities (206).

But interestingly, TIE2 signaling outcome can result in either a pro-inflammatory or an anti-inflammatory program according to the ligand engaged. ANG1 binding to TIE2 leads to receptor autophosphorylation on several tyrosine residues leading to signaling. Ang1 induces a pro-inflammatory phenotype in macrophages during differentiation, probably linked with P38 and ERK1/2 early activation but independent of AKT (207). However, TIE2-dependent phosphorylation of AKT prevented macrophages from apoptosis (208).

ANG2 was shown to convert macrophages to an anti-inflammatory or M2-polarized state (207, 209). Moreover, in cancer for example, when ANG2 is abundant, it binds to TIE2 and maintains TIE2 phosphorylation in an autocrine way, upregulates the expression of M2-type associated genes such as IL-10, Mannose Receptor C-Type 1 (MRC1) and CCL17 and pro-angiogenic genes such as thymidine phosphorylase (TP) and cathepsin B (CTSB) (209). However, ANG2/TIE2 interaction does not result in phosphorylation of the receptor, and instead, it acts as a competitive inhibitor preventing ANG1 binding.

##### G-protein-coupled receptors

Many extracellular signals are detected by GPCRs and transduced to heterotrimeric G proteins, which then transduce these signals intracellularly to suitable downstream effectors, playing a key part in numerous signaling cascades.

When activated by a ligand, GPCR proteins change conformation and subsequently activates the G proteins by increasing the exchange of GDP/GTP associated with the G subunit. This results in the dissociation of the G/G dimer from G. Both moieties are then free to engage on their downstream effectors and create distinct intracellular signaling responses.

**Figure 6:**
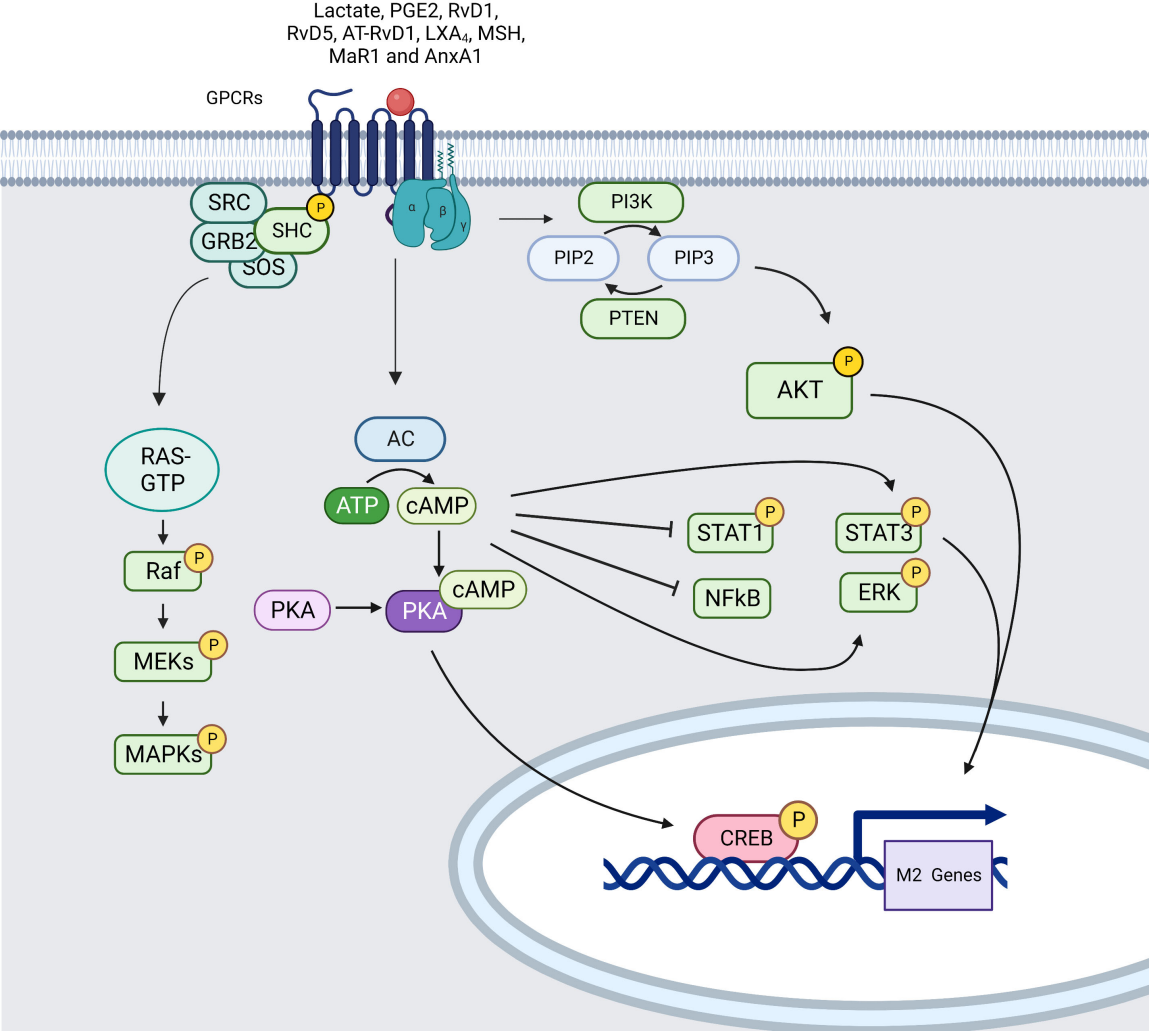
Simplified signaling pathway induced by GPCRs in macrophages (created using BioRender® ).

The activation of membrane receptors (mostly GPCRs) that activate cellular adenylyl cyclases (AC) converting ATP to cAMP, causes the generation of cAMP (210). As shown by using cAMP-inducing drugs, cAMP is able to decrease the secretion of TNF-α, IL-12, leukotriene B4 (LTB4), IL-1, and chemokines such as CCL3, CXCL1, CCL2, CCL4, and CCL11. cAMP also induces activation of STAT3 and STAT6 that lead to M2 polarization. In addition, activation of Epac1/2 by cAMP inhibits the production of pro-inflammatory cytokines through NF-κB pathway (211, 212) (**Figure 6**).

Several different mediators can lead to cAMP activation through GPCRs binding such as tumor-derived lactate (213), Resolvins (RvD1, RvD5 and AT-RvD1), lipoxins (LXA_4_), melanocortins (MSH), maresin 1 (MaR1), adenosine and potentially Annexin A1 (AnxA1) increasing AC activity through binding to GPCRs with subunits Gαs. Through activation of EP2–NF-κB signaling pathway, mediators such as PGE2 lead to cAMP induction triggering subsequent protein kinase A (PKA) activation inducing phosphorylation and nucleus translocation of cAMP-responsive element binding protein (CREB). CREB translocation promotes the production of anti-inflammatory cytokines and stimulate macrophage polarization. Moreover, PKA can inhibit NF-κB activity leading to diminution of inflammatory gene expression and activate ERK1/2 mediating the secretion of CCL2. cAMP drives M2 polarization by phosphorylating STAT3 and it also re-educates M1 macrophages towards an M2-like phenotype by lowering STAT1 phosphorylation *via* PKA (214, 215).

MAPK are also activated by ligands for heterotrimeric GPCRs mainly through the recruitment of GRB2/SOS complex that leads to RAS/RAF GTPase pathway activation and MEK/ERK signal transduction (216).

Chemokine receptors belong to the class A family of GPCRs and cluster of five chemokine receptors (CCR2, CCR5, CCR7, CX3CR1, and FPR1) is strongly expressed on myeloid cells. The chemokines CCL2, CXCL12 and the chemokine-like protein migration inhibitory factor (MIF) play a particular role in TAM polarization. Numerous models have dissected the pro-inflammatory axis between CCL2 and its corresponding receptor CCR2. In fact, blocking CCL2 during macrophage polarization upregulated the M1-associated HIF1α gene and enhanced the production of CXCL8 (217).

Sierra-Filardi et al. clearly demonstrate the anti-inflammatory intracellular signaling initiated by CCR2 ligation and indicate that CCL2 directs macrophage polarization toward the development of a M2 profile. This mechanism seems to be mediated through different pathways such as P38, HSP27 (an P38 downstream effector), ERK1/2, MSK1/2 (an ERK downstream kinase), JNK, STAT5a/b (218).

Another well studied chemokine in cancer is CCL5, which greatly promotes carcinogenesis, stroma formation, cancer progression and metastasis (219). Recent studies showed that CCL5 directly promotes M2 type polarization and that blocking CCL5-CCR5 binding led to M2 to M1 repolarization. Moreover, this modulation seems to appear through the JAK/STAT pathway (220). Inhibition of CCR5 (CCL5 receptor) using an antagonist antibody or drugs leads to repolarization of M2 to M1 tumor-associated macrophages (221, 222).

*Others GPCRs expressed in macrophages and leading to macrophage activation are referenced into Wang et al. review* (223).

##### SIRPα

The signal regulatory protein α (SIRPα)/CD47 axis has emerged as an important innate immune checkpoint that allows cancer cells to escape phagocytosis by macrophages. SIRPα is an immunoreceptor tyrosine-based inhibitory motif (ITIM)-containing receptor of the SIRP family expressed on all myeloid cell types including monocytes, macrophages, DCs, and neutrophils, and found to be strongly expressed in the TME. Extracellular ligation of SIRPα of its ligand CD47 alone does not significantly increase phosphorylation of SIRPα’s ITIMs but comes to counteract the activation induced by phosphorylation of ITAM-containing activatory receptors such as FcγRs.

A recent paper demonstrates that SIRPα signaling partially represses NF-κB, MAPK and STAT1 activation and potently inhibits PI3K-induced AKT2 activation in IFN-γ/LPS–treated macrophages leading to inhibition of pro-inflammatory M1 macrophage polarization. In parallel, in the same model, depletion of SIRPα induces overactivation of NF-κB, MAPK, and STAT1 pathways and moreover, SIRPα exposition to CD47 drastically decreases but also shortens the AKT2 phosphorylation through SHP1 recruitment (224).

In addition, another recent article showed that SIRPα and Notch Signal-Mediated Macrophage Polarization are probably linked. Indeed, Notch activation has been documented to repress SIRPα transcription directly through HES1-binding sites in its promoter region. Notch signal altered macrophage polarization in part by controlling the expression of SIRPα (225).

Indeed, SHP1 (PTPN6) and SHP2 (PTPN11) are paralog cytoplasmic PTPases that are crucial for a wide range of cellular functions. Through phosphotyrosine-based motifs such as ITIM and immunoreceptor tyrosine-based switch motif (ITSM), a significant number of inhibitory receptors like SIRPα recruit SHP1 and/or SHP2, tandem-SH2-containing phosphatases. SHP2 and SHP1 appear to be involved in a variety of signal transduction processes, such as the GRB2/Ras/Raf/MAPK, JAK/STAT, and PI3K pathways through direct interaction with signaling intermediates such as GRB2, FRS-2, JAK2, p85 subunit of PI3K, IRS1, GAB1 and GAB2 (226, 227).

Hence, by counteracting signaling cascades involved in pro-inflammatory responses, SIRPα may play a key role in modifying macrophage polarization in cancer in addition to its role as phagocytosis inhibitor.

##### Lilrb2

The leukocyte immunoglobulin-like receptor (LILR) family is a group of paired immunomodulatory receptors found in human myeloid and lymphoid cells. LILR subfamily A (LILRA) members connect with membrane adaptors to signal *via* ITAMs, whereas LILR subfamily B (LILRB) members signal *via* numerous cytoplasmic ITIMs (228). More interestingly, LILRBs are documented as being negative regulators of myeloid cell activation. Such as SIRPα, the ITIM domain of LILRB has the ability to bind SHP1/SHP2. Blocking antibodies targeting LILRB2 show reduction in receptor-mediated activation of SHP1/2 resulting in upregulation to pro-inflammatory pathways such as MAPK P38 and ERK, NF-κB or STAT1, and downregulation of AKT and STAT6 pathways leading to reprogramming towards a M1-like phenotype (229).

##### Egfr

The EGF receptor (EGFR), also known as ErbB1/HER1, is the prototype of the EGFR family, which also includes ErbB2/HER2/Neu, ErbB3/HER3, and ErbB4/HER4. With the ability to form homo- and heterodimers, these family members may construct a total of 28 distinct combinations with one another (230). The interaction of an EGF ligand-EGFR causes receptor dimerization, receptor trans-autophosphorylation on the C-terminal domain, and the recruitment of signaling proteins or adaptors. The phosphorylated C-terminal domain contacts SHC and GRB2. As described before, GRB2 SH3 domain recruits SOS or GAB1 proline-rich domains to initiate ERK MAPK or PI3K/AKT signaling, respectively. EGFR can also bind SCR and PLCy leading to activation of downstream well-known signals (231).

In addition to EGF-secreted action on tumor cell proliferation and survival, studies shows that secreted EGF also plays a crucial role in M2 polarization in cancer (232, 233).

## Conclusion and perspectives

TAMs have emerged as an interesting candidate population for innovative anti-tumor therapies and several emergent treatment approaches have been tested to reduce TAMs in tumors with, so far, limited efficacy. More recently, reprogramming M2-like TAMs into immunostimulatory and anti-tumor M1-like cells has appeared as an appealing approach in cancer therapy with encouraging preclinical and preliminary clinical data using antibodies targeting M2-like transmembrane proteins such as MARCO, CLEVER1 and ILT4 (234–236). Therefore, it is pivotal to better understand the mechanisms at the origin of the plasticity of this population.

In most of the cases, a stimulus from TME will trigger one or several of the JAK/STATs, MAPK, PI3K/AKT, NOTCH and NF-κB signaling pathways thus resulting in TAMs polarization. There is enough evidence establishing the association between M2 polarization and the activation of several signaling pathways including PI3K/AKT, JAK/STAT6 or STAT3, TGF-β/SMAD-dependent pathways. In addition, it is also possible to link directly JNK, P38, NF-κB p65, JAK/STAT1 signaling pathways and M1 polarization.

Indeed, the more currently advanced drugs targeting TAMs such as CSF1/CSF1R axis (237), MEK/STAT3 inhibitors (238), antibodies against IL-4, IL-4Rα, and IL-13 (239), IFN-γ (240, 241), CD40 agonists (242), inhibitors of PI3Kγ/mTOR (237) and agonists of TLR4/7/8/9 (237) all have an impact on macrophages signaling. For example, PI3Kγ inhibitor (ipi-549, phase II in combination with nivolumab), in addition to impact PI3K/AKT pathway will enhance drastically NF-κB phosphorylation after treatment (237). Moreover, patients with advanced malignancies are currently being tested with the STAT3 inhibitor (TTI-101) in a phase I clinical trial (NCT03195699). Indeed, targeting JAK2, the main activator of STAT3 in myeloid cells, is a crucial strategy for inhibiting STAT3. Another example is the use of anti-ILT4 mAbs, which was also found to activate P38, ERK, NF-κB and STAT1 while inhibiting the activation of STAT6 and AKT in the presence of M-CSF and IL-4 within 30 minutes of anti-ILT4 treatment (229). It has also been demonstrated that ILT4 blocking can both activate monocytes from PBMCs as well as acting in M2 macrophages to trigger M2-to-M1 reversion (229).

However, the M1 vs. M2 dichotomy is much less evident regarding other signaling pathways, such as NOTCH, ERK and even NF-κB, for which different studies demonstrated an implication in both polarization outcomes. Similarly, some receptors, which will be discussed further in the discussion section, can be associated with one phenotype, while others, depending on the stimulus, can lead to two different phenotypes. Moreover, some of them may operate a balance between the two phenotypes depending on time of exposure, timing of activation, specific serine/tyrosine phosphorylation, ligand type, multivalency and/or strength of stimuli in TME. Indeed, we cannot depict a black and white model that unequivocally defines whether the ligand-receptor engagement will be favorable to either M2 or M1 phenotype, and most likely, the integration of the different stimuli and the balance between their signals will determine the fate of macrophage polarization.

Besides, the analysis of TAMs using traditional, scRNA-seq, or time-of-flight (CyTOF) mass cytometry methods has shown the presence of several macrophage cell clusters with unique transcriptome and proteomic profiles. These methods have been essential in identifying the variety of TAMs outside the traditional M1-like or M2-like dichotomy in lung cancer, non-small cell lung cancer and brain tumors (243–245). Nevertheless, it remains clear that some sub-populations of TAMs either promote or limit cancer development and, as such, the M1-like and M2-like categories continue to have communicative value when the limitations of the macrophage classification are taken into account (246). A good illustration that could lead to better understanding of this paradoxes is the implication of signaling pathways that probably leads to more complex outcome than just M1/M2 dichotomy. However, the difficulty of characterizing different subpopulations of TAMs and of generating them *in vitro* under the extremely variableconditions of the TME has not yet made possible to study the involvement of signaling pathways in a more complex setting.

Additionally, this great heterogenicity in TAM could potentially explain limitations of targeting TAMs for tumor treatment. Indeed, in anti-CLEVER1 monotherapy, 7 of 30 patients with different pathology were classified as benefitting from the therapy by RECIST 1.1 (PR or SD response in target or non-target lesions) (247). Concerning anti-ILT4, clinical trial showed 1 partial response (PR) over 50 treated patients and 22% SD while anti-ILT4 in combination with pembrolizumab show encouraging results (21% PR, 26% SD and 3% complete response (CR)) (234, 235). This illustrates that targeting TAMs in combination with conventional immunotherapy treatments could significantly improve their effectiveness in the near future in the fight against cancer. Identification of TAM diversity at the single-cell level may open new perspectives on depletion strategy. This could justify the development of specific treatment against TAMs multiple subsets to improve clinic benefits of this kind of approaches.

## Discussion

One possible explanation for the ability of cell surface receptors to mediate opposite biological responses is that cell surface receptors may regulate signaling pathways quantitatively differently to mediate specific biological responses: low levels of receptor occupancy may result in low levels of receptor signaling, whereas high levels of receptor occupancy may result in high levels of receptor signaling. For example, in the case of the GM-CSF/GM-CSFR axis, the intrinsically activated signaling pathways vary depending on GM-CSF concentration (248). Indeed, a very low dose of GM-CSF seems rather to be at the origin of the activation of pathways such as PI3K/AKT/mTOR, whereas a higher dose could rather favor the JAK2/STAT5 and RAS/MAPK pathway. This phenomenon is directly linked with another relevant point regarding specific serine/tyrosine residues phosphorylation dynamics. Indeed, the activation of JAK2/STAT5 and ERK pathway by higher dose of GM-CSF requires Y577 phosphorylation thus providing a SHC-binding domain, whereas low doses of GM-CSF induce S585 phosphorylation thus providing a binding domain for p85 subunit of PI3K, leading to its recruitment and inducing PI3K/AKT/mTOR pathway activation.

Additionally, this duality of signaling pathways outcome depending on phosphorylation site is also illustrated by STAT3. Indeed, STAT3 is phosphorylated on Y705 after IL-10 stimulation of macrophages leading to M2 phenotype. In the opposite way, TLR4 stimulation by LPS can lead to STAT3 S727 phosphorylation inducing STAT3 mitochondrial translocation altering ROS production (129), thus favoring the polarization of pro-inflammatory macrophages.

There are also disparities due to the differential impact of kinase isoforms on phenotype outcome. Indeed, it is well known that PI3K activates AKT. However, it is unknown how the expression and activation of AKT isoforms are regulated in macrophages. PI3K/AKT pathway has convincingly been associated with M2 polarization, however, there are some evidences showing that PI3K/AKT pathway activation can lead both to M1 (PI3K/AKT2 activation) or M2 (PI3K/AKT1 activation) according to AKT isoform (169). There are also evidences that the activation of different PI3K isoforms may have an inverse impact on macrophage polarization, but further research on this topic would allow us to better understand these mechanisms (170).

Upregulation of HIFs in response to oxygen stress can also lead to opposite effects on macrophage polarization when involving HIF-1α or HIF-2α. Indeed, HIF-1α expression in macrophages is induced by Th1 cytokines such as IFNγ leading to M1 macrophage polarization, whereas HIF-2α is induced by Th2 cytokines leading to M2 polarization (66, 162).

The duration and timing of pathway activation has also been found to regulate pleiotropically the subsequent biological responses. For example, the ‘transient versus sustained’ MAPK ERK1/2 signaling can lead to diverse phenotypes. Indeed, while many studies show that ERK pathway is involved in the inflammatory response of macrophages leading to the secretion of IL-1β, TNF-α, IL-6 or advanced glycation end products (AGEs) (249), it has also been described to be activated during M2 polarization. Indeed, the duration of ERK activation can be either transient and short-lived, or sustained and lasting several hours (250). For example, it has been shown in human macrophages that IL-4 causes an increase in ERK1/2 phosphorylation between 2 and 8 hours but not between 10 and 30 minutes, leading to M2 polarization (249). Furthermore, stimulation of macrophages with M-CSF induces sustained activation of ERK1/2 (182), leading to the same outcome than IL-4 stimulation. In fact, early and rapidly diminishing activation of ERK1/2 was mainly associated with M1 promotion (249, 251–254), as opposed to late and more persistent activation that seems to lead to M2 phenotype (182, 249).

Notch and TGF-β are also a perfect example of signaling pathway bipolarity given NOTCH receptor binding to different ligands from DLL and JAG families, and the capacity of TGFβR to induce different pathways from the same ligand. On one hand, NOTCH/JAG1 and TGF-β canonical pathways favor M2 type polarization whereas NOTCH/DLL and TGF-β non canonical pathways seem to favor M1 polarization.

Another balanced mechanism concerns NF-κB signaling pathway. Many studies showed that NF-κB is activated by proinflammatory cytokines such as TNF-α, IL-1 and pathogen-associated molecular pattern molecules leading to expression of genes involved in immunological and inflammatory responses (e.g IL-1β, TNF-α, iNOS, ICAM-1, IL-12 and CCL2), due to the nuclear translocation of NF-κB p50/c-Rel or p65/p50 dimers (255). However, formation and nuclear accumulation of other dimers such as p50/p50 is essential for M2 polarization (256, 257).

Another interesting point is the existence of redundant or parallel signaling pathways. Indeed, these notions have been highlighted in the context of resistance to cancer therapies, especially when using drugs against proliferative pathways. “Parallel” signaling pathways are defined as functionally and evolutionarily distinct, such as Notch, JAK, PI3K and MAPK, but are all capable of promoting cell proliferation. These signaling pathways are commonly considered redundant as they can fulfil the role of cell proliferation by substituting for the original pathway that has been repressed. Indeed, when drugs block one pathway, resistance may arise through the activation of other signaling pathways that are redundant or parallel. Furthermore, redundant signaling pathways are defined by the use of the same downstream signaling targets, such as K-RAS, H-RAS and N-RAS that can all active the MEK/ERK pathway (258). However, given the enormous diversity of stimuli present in the TME, which drive macrophage plasticity through the activation/repression of many different signaling pathways, this redundancy may play a key role in macrophage polarization and should attract our attention for a better understanding of these mechanisms and better thoughts on therapeutic approaches.

All these observations show that the plasticity of macrophages is tightly linked to the balance between the activation and inhibition of many different signaling pathways and remind us that a deep understanding of these mechanisms must be coupled with phenotype and functional characterization for macrophage polarization understanding and subsequent development of potent cancer therapies.

## Author contributions

CK conceived of and wrote the review. CEC and DO reviewed the review. All authors contributed to the article and approved the submitted version.

## Acknowledgments

This work was sponsored by ImCheck Therapeutics and CRCM. We thank our colleagues from ImCheck Therapeutics for providing insight and knowledge that substantially helped for writing the review. We gratefully acknowledge, Etienne Foucher, Sophie Agaugué and Loui Madakamutil for suggestions that significantly improved the text as well as Aude De Gassart for assistance with figure conception.

## Conflict of interest

CK and CEC are ImCheck Therapeutics employees. DO is a co-founder of ImCheck Therapeutics, Alderaan Biotechnology, and Emergence Therapeutics and has research funds from ImCheck Therapeutics, Alderaan Biotechnology, Cellectis, and Emergence Therapeutics.

## Publisher's note

All claims expressed in this article are solely those of the authors and do not necessarily represent those of their affiliated organizations, or those of the publisher, the editors and the reviewers. Any product that may be evaluated in this article, or claim that may be made by its manufacturer, is not guaranteed or endorsed by the publisher.
